# Contributing to collaborative health governance in Africa: a realist evaluation of the Universal Health Coverage Partnership

**DOI:** 10.1186/s12913-022-08120-0

**Published:** 2022-06-06

**Authors:** Emilie Robert, Sylvie Zongo, Dheepa Rajan, Valéry Ridde

**Affiliations:** 1 École de santé publique de l’Université de Montréal, 7101 Avenue du Parc, Montreal, QC H3N 1X9 Canada; 2 ICARES, Montreal, QC Canada; 3 Centre de Recherche SHERPA, Montreal, QC Canada; 4 Institut des Sciences des Sociétés (INSS), Centre National de la Recherche Scientifique et Technologique du Burkina Faso, Ouagadougou, Burkina Faso; 5 Department of Health Systems Governance and Financing, World Health Organization, Geneva, Switzerland; 6 Institut de recherche pour le développement (IRD), Paris, France

**Keywords:** Policy dialogue, Health, Governance, Africa, Collaboration, Development aid, Realist evaluation

## Abstract

**Background:**

Policy dialogue, a collaborative governance mechanism, has raised interest among international stakeholders. They see it as a means to strengthen health systems governance and to participate in the development of health policies that support universal health coverage. In this context, WHO has set up the Universal Health Coverage Partnership. This Partnership aims to support health ministries in establishing inclusive, participatory, and evidence-informed policy dialogue. The general purpose of our study is to understand how and in what contexts the Partnership may support policy dialogue and with what outcomes. More specifically, our study aims to answer two questions: 1) How and in what contexts may the Partnership initiate and nurture policy dialogue? 2) How do collaboration dynamics unfold within policy dialogue supported by the Partnership?

**Methods:**

We conducted a multiple-case study realist evaluation based on Emerson’s integrative framework for collaborative governance to investigate the role of the Partnership in policy dialogue on three policy issues in six sub-Saharan African countries: health financing (Burkina Faso and Democratic Republic of Congo), health planning (Cabo Verde, Niger, and Togo), and aid coordination for health (Liberia). We interviewed 121 key informants, analyzed policy documents, and observed policy dialogue events.

**Results:**

The Partnership may facilitate the initiation of policy dialogue when: 1) stakeholders feel uncertain about health sector issues and acknowledge their interdependence in responding to such issues, and 2) policy dialogue coincides with their needs and interests. In this context, policy dialogue enables stakeholders to build a shared understanding of issues and of the need for action and encourages collective leadership. However, ministries’ weak ownership of policy dialogue and stakeholders’ lack of confidence in their capacity for joint action hinder their engagement and curb the institutionalization of policy dialogue.

**Conclusions:**

Development aid actors wishing to support policy dialogue must do so over the long term so that collaborative governance becomes routine and a culture of collaboration has time to grow. Public administrations should develop collaborative governance mechanisms that are transparent and intelligible in order to facilitate stakeholder engagement.

## Background

Governance in low- and middle-income countries (LMIC) has been the focus of considerable literature, especially since good governance became a development goal [1, 2]. This topic has gained so much interest that some speak of a “governance market” [3]. In the health sector, governance is supported by global health actors [4–6], many of whom are involved in global health initiatives [7]. The goal is to strengthen the accountability of public authorities, the engagement and coordination of actors in the system, and transparency in decision-making [8]. Such progress should help to improve the management and functioning of health systems, most of which, particularly in sub-Saharan Africa, have large room for improvement, especially when they need to go into emergency management mode [9, 10]. As a critical element of the health system [11], governance includes “making, changing, monitoring and enforcing the rules that govern the demand and supply of health services” [12]. Governance further affects the health system’s capacity to respond to challenges and ensure the sustainability of quality health services universally accessible [8]. Efforts to strengthen health system governance, through establishing partnerships and opening concertation spaces to multiple public and private stakeholders, are expected to contribute to universal health coverage by improving the performance of health systems [13].

In this context, WHO set up the Universal Health Coverage Partnership (the Partnership). It aims to support policy dialogue, a collaborative governance instrument, in countries working towards universal health coverage (UHC) [14]. UHC requires joint efforts from a multitude of actors (including government institutions, international and development aid organizations, civil society and the private sector). For that purpose, WHO and its partners consider policy dialogue as an efficient way for generating the policy documents needed for UHC and for strengthening collaboration dynamics. Policy dialogue is defined as “a collaborative instrument for multi-stakeholder governance of health” [15]. The objective of the Partnership is to promote evidence-informed, inclusive and participatory policy dialogue led by health ministries. WHO’s support consists of technical assistance of varying intensity and duration depending on the country. It may include the deployment of international policy dialogue experts, as well as health financing, health planning or health system experts. This technical assistance is complemented by financial resources used primarily for organizing concertation spaces. The Partnership therefore works to provide health ministries with tools for collaborative governance and instil a culture of collaborative dialogue among stakeholders, with the view to improve governance of the health sector. Stakeholders’ involvement in policy dialogue are expected to contribute to increased buy-in of decisions, greater trust and a better understanding of interests, values and needs among them.

WHO plays several roles in the Partnership. First, WHO acts as a technical adviser when it supports structured and transparent policy dialogue, and helps strengthen health ministries’ leadership and management functions. Second, WHO plays a brokering role when it contributes to evidence-informed policy dialogue and helps to identify workable compromises that take equity into account. Third, WHO is a driving force when it is able to generate synergy among policy dialogue stakeholders. The Partnership is, therefore, a multifaceted program adapted to countries’ needs, sensible to contextual influences, and contributing to various potential outcomes.

The general purpose of our study is to understand how and in what contexts the Partnership may support policy dialogue and with what outcomes. More specifically, our study aims to answer two questions: 1) How and in what contexts may the Partnership initiate and nurture policy dialogue? 2) How do collaboration dynamics unfold within policy dialogue supported by the Partnership?

## Methods

### The conceptual framework

We conducted a multiple-case study realist evaluation of the Partnership based on Emerson's integrative framework for collaborative governance [16]. Our study uses a realist approach as the ontological and epistemological foundations [17]. According to this approach, an intervention – such as the Partnership – produces outcomes in certain contexts, through the triggering of mechanisms. This is called generative causation. A mechanism is defined as the reasoning or reaction of actors regarding the resources an intervention makes available to them [18]. In a realist evaluation research, the objective is to provide a theoretical explanation of how the intervention produces outcomes in particular contexts through the triggering of one or more mechanisms. Researchers seek to identify regular, but not necessarily systematic, interactions between an intervention, mechanism, outcome, and context. These interactions, also known as context-intervention-mechanism-outcome (CIMO) configurations, become demi-regularities when they are identified on several occasions. We propose demi-regularities to better understand the role of the Partnership as the results of this evaluation.

We combined the realist approach with the integrative framework for collaborative governance from Emerson et al. [16, 19] to understand the role of the Partnership in relation with policy dialogue. According to Emerson et al., collaborative governance refers to “the processes and structures of public policy decision making and management that engage people across the boundaries of public agencies, levels of government and/or the public, private and civic spheres to carry out a public purpose that could not otherwise be accomplished” [19]. Policy dialogue is an example of collaborative governance:

• It shapes public decision-making in health;
• It consists of discussions and negotiations leading to the drafting of policy documents (e.g., national health financing strategies); and
• It relies on the collaboration of stakeholders, regardless of their area of activity (health, finance, civil service, social protection, etc.) and nature (public, private, semi-public, civil society).

In Emerson’s integrative framework, collaborative governance is broken down into three stages: collaboration initiation, which is impacted by several drivers triggering the collaboration, collaboration dynamics that require resources, and finally, collaborative actions that may generate positive feedback loops. This process takes place in a context that influences how collaboration unfolds, which Emerson calls the system context: “This system context generates opportunities and constraints and influences the dynamics of the collaboration at the outset and over time.” [16]. The six components of the system context are: resource or service conditions, policy and legal frameworks, socioeconomic and cultural characteristics, network characteristics, political dynamics and power relations, and history of conflict.

One of the drivers of collaborative governance is leadership, i.e., the need for an actor to “initiate and help secure resources and support” for a collaborative governance regime [16]. The Partnership plays such a role in the policy dialogue initiatives towards UHC, which explains our choice to use Emerson’s framework. Furthermore, the dimensions of Emerson’s framework can be operationalized using the realist approach, as several of them are contextual dimensions (e.g., system context), while others are generative mechanisms (e.g., principled engagement).

Emerson et al. put forward several propositions about how the framework’s components interact [16]. We used five of these propositions to analyze the role of the Partnership and uncover regular occurrences of interactions between the Partnership, context, mechanism and outcomes. Emerson’s propositions allow to gain a full picture of the Partnership’s many contributions to policy dialogue. They also help with identifying the different contexts that impacted the Partnership’s ability to carry out its role and determining whether policy dialogue was possible.

- Proposition on the drivers of policy dialogue: “One or more of the drivers of leadership, consequential incentives, interdependence, or uncertainty are necessary for (policy dialogue) to begin. The more drivers present and recognized by participants, the more likely (policy dialogue) will be initiated.” (p.10)
- Proposition on principled engagement: “Principled engagement is generated and sustained by the interactive processes of discovery, definition, deliberation, and determination. The effectiveness of principled engagement is determined, in part, by the quality of these interactive processes.” (p.13)
- Proposition on shared motivation: “Repeated, quality interactions through principled engagement will help foster trust, mutual understanding, internal legitimacy, and shared commitment, thereby generating and sustaining shared motivation.” (p.14)
- Proposition on capacity for joint action: “Principled engagement and shared motivation will stimulate the development of institution arrangements, leadership, knowledge, and resources, thereby generating and sustaining capacity for joint action.” (p.16)
- Proposition on collaborative actions: “Collaborative actions are more likely to be implemented if 1) a shared theory of action is identified explicitly among the collaboration partners and 2) the collaborative dynamics function to generate the needed capacity for joint action.” (p.18)

### Study design and case description

We carried out this realist evaluation in six countries in sub-Saharan Africa. We published the research protocol [20] and a pedagogical case study where we account for methodological challenges and lessons learned [21]. The following elements of the methods are explained in details in the research protocol: intervention theory, case selection and sampling methods, realist analytical procedures, justification of internal and external validity.

Our study includes five low-income countries (Burkina Faso, Liberia, Niger, Togo, and DRC) and one middle-income country (Cabo Verde) in Africa. We used purposive sampling to select the countries and contrasted sampling for the policy dialogue initiatives. We investigated the role of the Partnership in six policy dialogue processes on three topics: health financing (Burkina Faso and DRC), health planning (Cabo Verde, Niger, and Togo), and aid coordination for health (Liberia).

We performed a qualitative comparative descriptive analysis of all the policy dialogue initiatives. In the analysis, we rated four dimensions on a scale from 0 to 4 (Table 1) to provide readers with a frame of reference for the cases. Each dimension represents a characteristic of policy dialogue. Spider diagrams illustrate the policy dialogue experiences which are categorized according to the level of support provided by the Partnership.

**Table 1:** Dimensions and scores for the comparative analysis of policy dialogue initiatives

Level of Partnership support
1. No support from the Partnership, but WHO is present
2. Part-time expert
3. Full-time expert
4. Full-time expert + full-time consultant
**Level of interest in the ministry**
1. No support; presence is symbolic in meetings
2. Experts are involved but passive, and have no particular interest in the policy dialogue
3. Experts are interested and involved, but no decision-makers are involved
4. Experts and decision-maker(s) are involved
**Level of national interest**
1. Symbolic interest in policy dialogue (mainly in documents and discourse)
2. General interest in UHC
3. Keen interest in policy dialogue (active participation of important ministries)
4. Policy dialogue on the government’s agenda
**Dialogue momentum**
1. Overall lack of attendance at meetings, which are difficult to hold
2. Stakeholders are repeatedly absent; multisectorality and inclusion is weak
3. Stakeholders are mostly involved in drafting committees, but others (at the strategic level) are difficult to convene
4. Vigorous process involving all participants

### Data collection and analysis

Data collection took place throughout 2017, except in Togo, where it began in 2016 as part of a pilot study to test the approach and methods. We conducted semi-structured interviews with key informants, including policy dialogue participants and representatives of organizations involved in the policy dialogue (*n* = 121), analyzed policy documents, and observed policy dialogue events in Burkina Faso and Cabo Verde. Table 2 presents the number of respondents and observation opportunities by case and indicates the types of analyses conducted.

**Table 2:** Description of data collection and types of analysis

Case	Burkina Faso	Cabo Verde	Liberia	Niger	Togo	DRC
Number of respondents	*N* = 29	*N* = 28	*N* = 11	*N* = 29	*N* = 29	*N* = 24
Observation opportunities	YES	YES	NO	NO	NO	NO
Chronology of policy dialogue	Complete	Complete	Partial	Partial	Complete	Partial
Document analysis	*N* = 26	Not available	*N* = 17	*N* = 4	*N* = 13	*N* = 11
Stakeholder analysis	Complete	Complete	Partial	Complete	Complete	Complete
Analysis of barriers and facilitators	Complete	Not available	Partial	Not available	Complete	Complete
Realist analysis	Partial	Not available	Partial	Partial	Complete	Complete

We performed five types of analyses, to various extents depending on the availability and quality of data and expertise, and on the researchers’ availability in each of the six countries. Since we could not perform a realist analysis in some case studies, we were unable to conduct the transversal analysis using CIMO configurations, as set out in the protocol. As an alternative, we adopted a realist synthesis approach [22], which allowed us to use the configurations from realist cases (Burkina Faso, Niger, Togo, and Democratic Republic of the Congo (DRC)), and integrate empirical data from other cases (Cabo Verde, Liberia) to confirm, contradict or specify the transversal demi-regularities. The data either helped to establish demi-regularities by showing one or more interactions between context, mechanism, and outcome, or provided counterexamples, indicating an absence of interaction. The demi-regularities highlighted in the following sections are, therefore, theoretical propositions.

Each case study has strengths and weaknesses, and the quantity and quality of data and depth of the analyses vary. Performing realist analyses of the cases was thus a significant challenge, and some were more comprehensive than others. The realist synthesis was possible owing to the wealth of empirical data. Another limitation concerned our real-time access to policy dialogue debates, discussions, and negotiations. As opportunities for in situ observations were limited, we were unable to uncover some of the critical decision-making processes, and scrutinize the nature of interactions between stakeholders. Identifying these elements in the interviews was also tricky. Lastly, concerning contextual dimensions, stakeholder networks were not investigated beyond a traditional stakeholder analysis.

## Results

We first present the characteristics of the policy dialogues initiatives. We then describe some key elements from the general system context affecting collaboration dynamics and the Partnership’s ability to initiate and support policy dialogue. We continue by proposing eight demi-regularities that take the form of context-(intervention)-mechanism-outcome propositions, in three distinct sections corresponding to three stages of collaboration: policy dialogue initiation, policy dialogue nurturing, and policy dialogue dynamics.

### Characteristics of policy dialogue initiatives

Policy dialogue in Burkina Faso, Niger, Togo, and DRC was collaborative, while it was consultative in Cape Verde, where it was led by external consultants, and in Liberia, where it involved more consultations than collaborative processes. WHO provided a full-time international policy dialogue expert in Liberia, Togo, and DRC, and a part-time expert in Burkina Faso, Cabo Verde, and Niger. Togo was a particular case since WHO provided both an international expert and a consultant at the country level to support policy dialogue on a full-time basis. Burkina Faso was also unique in that the policy dialogue received constant support from an international health financing expert. Table 3 provides a summary of the policy dialogue initiatives and characteristics of the Partnership.

**Table 3:** Summary of the policy dialogue initiatives and characteristics of the Partnership

Case	Burkina Faso	Cabo Verde	Liberia	Niger	Togo	DRC
Policy dialogue	Health financing	Regional health planning	Aid coordination	National health planning	National health planning	Health financing
Approach	Collaborative	Consultative	Consultative	Collaborative	Collaborative	Collaborative
Technical assistance	Part-time (light mode)	Part-time (light mode)	Full-time (full mode)	Part-time (light mode)	Full-time (full mode)	Full-time (full mode)

**Fig. 1:**
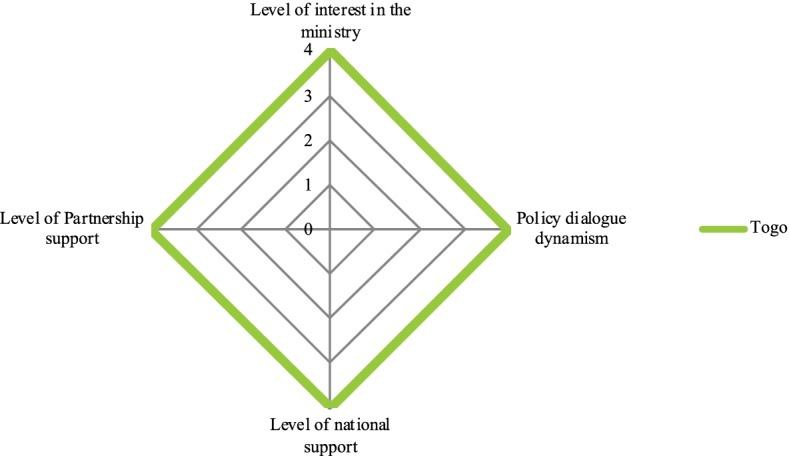
Policy dialogue on health planning in Togo

Togo’s experience (Fig. 1) was an outlier: as acknowledged by all stakeholders, there was strong momentum for health planning policy dialogue, which became a government priority. Togo appears a unique experience where the Partnership provided a high level of support, unlike in any other country, and where the Togolese health ministry’s decision-maker got actively involved and committed.

**Fig. 2:**
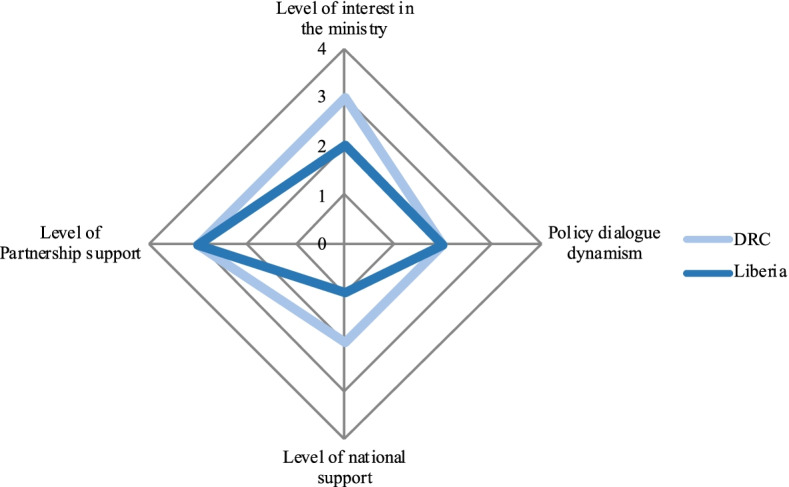
Policy dialogue on health financing in DRC and on aid coordination in Liberia

**Fig. 3:**
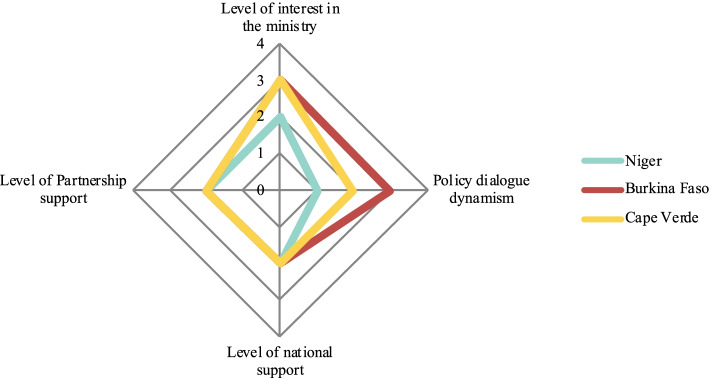
Policy dialogue on health financing in Burkina Faso and on health planning in Niger and Cabo Verde

In Liberia and DRC (Fig. 2), there was a relatively moderate level of momentum for the policy dialogue, which received more or less interest among stakeholders, despite the presence of a full-time international expert from the Partnership. Lastly, in Cabo Verde, Niger, and Burkina Faso (Fig. 3), the Partnership provided comparatively less support, as there was no full-time policy dialogue international expert. In those countries, policy dialogue was more or less dynamic. Burkina Faso stood out in that regard, possibly because an international health financing expert was present throughout and stimulated the policy dialogue.

### General system context affecting collaboration dynamics and the Partnership’s ability to initiate and support dialogue

Certain contextual conditions limit or facilitate the implementation of policy dialogue. Although not decisive, these conditions may have influenced the Partnership’s ability to initiate and support policy dialogue, as well as policy dialogue dynamics. The systemic conditions described below are not exhaustive; they were identified during the research or in the relevant literature.

#### Resource or service conditions

Weak health indicators demonstrate that Niger, Burkina Faso, DRC, Togo, and Liberia have dysfunctional and underfinanced health systems. These indicators have prompted global health actors to participate in several global health initiatives [23], including the H6 + and the Global Financing Facility. Confronted with the “medical poverty trap” and catastrophic health expenditures [24], these countries announced free health care policies, the implementation of which remains a challenge [25]. Such policies are considered a step towards UHC, a global health priority [26]. In this conducive environment for UHC initiatives, policy dialogue was initiated, in particular on health financing in Burkina Faso and DRC. Financing issues remain, indeed, the main focus of talks on UHC [27, 28].

In these countries, many global health actors [29] are working alongside health ministries to improve the health and well-being of communities. They seek to overcome the deficiencies of public health administrations or build their capacities. Their presence, yet, makes the health system landscape more complex. Coordination and alignment between their actions and health ministries’ objectives are usually lacking. In response to the growing interest for UHC and the countries’ health system deficiencies, global health actors are calling for multisectoral collaboration [15], paving the way for collaborative health governance mechanisms such as policy dialogue. The large number of actors involved at the national level poses, however, a challenge to identifying the various stakeholders and mobilizing them for policy dialogue. Although they have similar objectives, they have their institutional logics and organizational values that may conflict with one another and affect their openness to policy dialogue [15].

As a middle-income country, Cabo Verde is unique: compared with the other countries of the study, its health system is better, and its health ministry is more experienced. As an example, the share of public spending on health is higher [30]. Cabo Verde is experiencing an epidemiological transition, the leading cause of mortality being non-communicable diseases [31]. Regionalization, which is among the health ministry’s priorities, aims to give more autonomy to insular institutions in the health sector [32] and is creating expectations among regional stakeholders. Serving on municipal health commissions, these stakeholders want to be actively involved in initiatives for developing health-related competencies at the regional level.

#### Policy and legal frameworks

In most countries studied, ministerial orders have provided for multisectoral collaboration among decision-making bodies in the health sector. Examples are Niger’s national health committee and national technical health committee, which are in charge of managing and monitoring health development plans, and Togo’s HIV/health committee, which was in charge of coordinating and managing the implementation of the national plan for health development from 2011. In DRC, the 2009 ministerial order on the creation of the national health steering committee and its various technical committees provides for the participation of other sectors: it stipulates that the committee’s chairperson may invite to a meeting any person or institution whose presence is deemed necessary [33], including representatives of related ministries. In Cabo Verde, municipal health commissions have been established at the regional level and include representatives of civil society, health promotion associations, unions, and public entities from the health, education, and environment sectors.

All the countries studied are part of the International Health Partnership (IHP +). Now the International Health Partnership for UHC 2030 (UHC2030), this partnership promotes the coordination of all health actors, including civil society and the private sector, according to the Paris Declaration [34]. As signatories, governments signed a health Compact outlining each partner’s commitments to achieving common national objectives, including UHC. Although not legally binding, these numerous commitments promote multisectoral collaboration as a means to improve health sector coordination and outcomes. It is, however, impossible to predict whether the structures and commitments set out in instruments like health Compacts will be implemented thoroughly [35].

#### Socioeconomic and cultural characteristics

Niger, Burkina Faso, DRC, Togo, and Liberia are low-income countries. In addition to health challenges, they face endemic poverty, particularly in rural areas, and income per capita is low. Although government officials generally have a stable income, they are paid less than most of their collaborators in public international organizations or international non-governmental organizations [35]. This situation contributes to brain drain non only to foreign countries, but also from public administrations to development aid organizations or the private sector [36]. WHO is no exception. This context makes it difficult to identify collaborators who are qualified and available to contribute constructively to policy dialogue on a long-term basis or participate in a “purposeful manner” [16]. Faced with financial challenges, many people also try to maximize their income in the so-called race for per diem payments [37], notably via participating in capacity-building seminars [38], which affects stakeholders’ participation in policy dialogue. Cabo Verde is a middle-income country. Its transition from low- to middle-income status has led to a decrease in development aid and has raised concerns that the country will fall into the middle-income trap [39] while income inequalities and health challenges persist [40]. This context is also affected by human and financial resource issues, which influence partners’ level of involvement in policy dialogue.

#### Political dynamics and power relations

Identifying power dynamics within the countries is arduous since they are complex and are at play across several subsystems. We will give an overview of them based on findings from other studies, except for political dynamics, which we will not address. Concerning the overall development aid system, power dynamics in low-income countries generally give the most advantage to donors [41–44], in particular to those with significant economic capital [45]. This situation is mainly due to countries’ financial needs, in particular in the underfinanced health sector. As a result, external resources for health are a financial windfall. In the countries studied, in 2016, external resources accounted for more than 20% of total health spending, except for Niger and Cabo Verde, and more than 40% in DRC [46]. Often because of their financial weight or history, as is the case with USAID in Liberia, donors enjoy significant symbolic power and can mobilize highly skilled international experts to support countries. In comparison, less power is held by civil society, in particular local non-governmental organizations.

In the health subsystem, WHO has symbolic power [45], particularly in western Africa, where it is the "official adviser" to the health ministry. Since its funding has decreased in recent decades [47], its economic capital is weaker than that of several global health actors, including global health initiatives (e.g., Gavi), international institutions, (e.g., the World Bank), or even philanthropies, such as the Gates Foundation, which became one of its donors. Power relations among global health actors are thus linked to their capacity to mobilize financial resources, which relies on their capacity to demonstrate the effectiveness of the aid they provide. Some observers refer to “flag politics” (“politique des drapeaux”) [43], indicating that donors work hard on identifying projects that they finance and taking credit for their success. The sector-based approach, which has replaced the project-based approach, somewhat limits this mindset since funding is increasingly either pooled or provided jointly, making it difficult to claim credit for results [44].

National institutions are impacted in different ways in this system and make up a subsystem. In this subsystem, certain ministries have more power than others, in particular ministries of finance and the economy. Ministries in charge of social issues, including health ministries, often lack qualified human resources and are often underfinanced.

In Cabo Verde, governance has improved [40], and the health care system has become less complicated, involving fewer development aid actors, since Cabo Verde became a middle-income country. In this context, WHO has continued to engage with the health ministry, mainly to support decentralization. Several actors are involved in the health sector at the regional level, where municipal commissions are gaining autonomy. However, the central government remains very active [40].

This overview demonstrates how collaboration dynamics and decision-making processes in policy dialogue can vary according to countries’ contexts and the stakeholders involved.

#### History of conflict between key actors and organizations

Apart from Cabo Verde, which is among the top-scoring half of countries on the index for perceived corruption, the countries studied are among the lowest-scoring half on the index, with DRC at the very bottom [48]. Corruption in public administrations is seen not only as a barrier to public trust in institutions but also as an element that determines the nature of relations with donors [43].

Armed conflict and sociopolitical and health crises are not included in Emerson’’s framework but also deserve attention. In Liberia, successive corruption-related conflicts have shaped the development aid landscape, relations between its actors, and the population’s way of life. They have prevented donors and the State, which has essentially been illegitimate during certain periods [49–51], from trusting one another [52]. As a result, development aid actors have intervened outside of the often absent institutional framework, which has impeded collaboration. Besides, the recent Ebola outbreak led to fierce criticism of development aid actors [53, 54] and made coordination difficult as new organizations appeared in the health sector, bringing new financial manna. In Togo, various sociopolitical crises have led to self-censorship, making policy dialogue a challenging undertaking. This context has been hardly conducive to multisectoral collaboration.

To summarize, in all the countries except for Cabo Verde, we found several systemic conditions conducive to policy dialogue. The most evident among them were the deficiencies of health systems, the need to work collectively towards UHC, attempts by decision-making bodies in health to be open to multisectoral action. Systemic conditions impeding policy dialogue could include the countries’ socioeconomic status, which resulted in less stability and involvement of local human resources; the multitude of actors involved, which made mobilization and decision-making complex; sectoral responsibilities and power issues, which prevented multisectoral collaboration; and corruption and conflict, which created distrust among partners. In Cabo Verde, good governance, a lower number of development aid actors, decentralization, and municipal health commissions were systemic conditions favorable to policy dialogue.

In the following three sections, we propose eight demi-regularities that take the form of context-(intervention)-mechanism-outcome propositions, according to three stages: collaboration initiation, collaboration nurturing, and policy dialogue dynamics. Table 4 present all eight demi-regularities.

**Table 4:** Summary of demi-regularities

Demi-regularities
**The Partnership’s role in the initiation of policy dialogue**
(1) The Partnership facilitates the initiation of policy dialogue (O) when it financially supports stakeholders’ participation (I), because it aligns with the per diem payments culture (M) in low-income countries, where citizens seek to maximize their income (C).
(2) The Partnership facilitates the initiation of policy dialogue (O) when the opportunities for multisectoral exchange that it stimulates (I) respond to the needs and interests of relevant stakeholders (M) in situations involving external pressure (C).
(3) The Partnership facilitates the initiation of policy dialogue (O) by generating interest in multisectoral collaboration among stakeholders (M), provided that the latter acknowledge their interdependence and the uncertainty for managing essential health issues (C).
**The Partnership’s role in nurturing policy dialogue**
(4) The Partnership promotes principled engagement among policy dialogue stakeholders (O) through facilitating knowledge generation and providing tailored technical expertise (I), which enable stakeholders to gain a shared understanding of issues and acknowledge the need for collective action (M), provided that they understand the policy dialogue process and see the added value of their contribution (C).
(5) When health ministries are dynamic and engaged (C), the Partnership encourages stakeholders' commitment to policy dialogue (O) by promoting collective leadership in key positions (M). Collective leadership increases participants' involvement and motivation (O) owing to the symbolism associated with decision-makers' hierarchical positions (M) and with reciprocity (M).
**Policy dialogue dynamics**
(6) In the context of commodification of meeting opportunities (C), weak ownership of policy dialogue by health ministry decision-makers creates an adverse environment that discourages stakeholders (M) and reduces their participation (O), despite the Partnership’s support (I).
(7) In a context of collective leadership (C), full-time international experts (I) promote ownership over policy dialogue processes (O) by responding to the needs of their ministerial counterparts and by helping them to establish and monitor policy dialogue (M), which contributes to the institutionalization of multisectoral collaboration (O).
(8) In contexts where the health ministry demonstrates weak leadership (C), policy dialogue is unlikely to foster collaboration of stakeholders for the implementation of collective decisions (O) since policy dialogue participants lack confidence in their capacity for joint action and the ministry's abilities to take its stewardship role (M).

*C* context, *I* intervention, *M* mechanism, *O* outcome

### The Partnership’s role in the initiation of policy dialogue

The Partnership’s ability to raise stakeholders’ interest in policy dialogue depends on internal and external drivers that impact the triggering of mechanisms for collaborative governance. The following demi-regularities demonstrate the impact of such drivers or incentives.

#### Financial support as a necessary incentive in low-income countries (demi-regularity 1)

*The Partnership facilitates the initiation of policy dialogue (O) when it financially supports stakeholders’ participation (I), because it aligns with the per diem payments culture (M) in low-income countries, where citizens seek to maximize their income (C).*

This configuration was found in all the countries except for Cabo Verde. The Partnership provides new financial resources that allow WHO to fund policy dialogue. These funds can be used to rent premises, provide accommodation, meals, and breaks, and reimburse participation costs through per diem payments, in particular for workshops taking place outside of capital cities. Per diem payments can also be taken over by health ministries when meetings occur in capital cities, or by other development aid organizations when policy dialogue is jointly organized. In Burkina Faso, Niger and Togo, such external consequential incentives, in particular per diem payments, helped to mobilize participants in policy dialogue. As an extrinsic motivator, per diem payments act as "participation booster" [55], even more in the context of "per diem payments culture" [37]. However, they can also have adverse effects [37, 55]. In the race for per diems, people pursue the most lucrative activities to maximize their income. This strategy was observed in Burkina Faso, where the health ministry nor the Partnership did not systematically pay for per diems, which translated into low participation. These key resources of the program are therefore necessary, but insufficient. Participation often decreases in policy dialogues that span several years, like those in Burkina Faso and Niger (see demi-regularity 6).

#### Policy dialogue as a response to stakeholders’ needs and interests (demi-regularity 2)

*The Partnership facilitates the initiation of policy dialogue (O) when the opportunities for multisectoral exchange that it stimulates (I) respond to the needs and interests of relevant stakeholders (M) in situations involving external pressure (C).*

This configuration was found in Togo, DRC, and Burkina Faso. Stakeholders’ interests and needs are consequential internal incentives that influenced their participation in collaborative, multisectoral processes. These interests and needs vary according to the stakeholders involved (ministerial collaborators, representatives of civil society or the private sector, development aid organizations), and the external pressures at play. In Burkina Faso, prompted by the international and national context, several development aid organizations had prior interests regarding health financing and UHC. A national financing strategy was needed owing to the fragmentation of the health financing system and concerns that funds were being misused. Collaborators from the health ministry were also interested in health financing because national initiatives considered as health financing strategies (for example, user fee exemption policies and results-based financing) were already in place. Besides, Burkina Faso’s government was introducing universal health insurance, putting external pressure on the health ministry and their development partners. Providing financial support and international technical assistance on health financing, in collaboration with P4H, the Partnership thus met stakeholders’ expectations. In Togo, the Partnership had proven useful to the decision-maker in charge of health planning, who had recently taken up the position with limited experience in the area. The Partnership happened to be a significant external incentive as it provided the necessary technical and financial support. In DRC, although the health ministry had prior interest in UHC and health financing, it was the national assembly, in rejecting the public health act, that urged for the collaboration of the health ministry with other sectors committed to UHC, specifically within the framework of the new national policy on social protection. These findings confirm Ribesse’s propositions on technical assistance [56], which posit that technical assistance interventions have to be aligned with the priorities of the ministries being supported to help them reach their objectives. In these contexts, the Partnership provides an opportunity which partners, in particular health ministries, may take advantage of in order to implement or strengthen collaborative governance tools. However, initial interest in policy dialogue may fade over time as ministerial decision-makers face other priorities (see demi-regularity 6).

#### WHO as a proponent of multisectoral collaboration (demi-regularity 3)

*The Partnership facilitates the initiation of policy dialogue (O) by generating interest in multisectoral collaboration among stakeholders (M), provided that the latter acknowledge their interdependence and the uncertainty for managing essential health issues (C).*

We observed this configuration in Togo and Liberia. In Togo, technical and financial partners followed WHO’s recommendation for a joint evaluation of the 2012–2015 national health development plan (NHDP), taking a collaborative approach. They saw it as an opportunity to contribute to health planning and to pursue the commitments made under a moribund Compact, in a context marked by external pressures and several uncertainties. Such uncertainties included the regional Ebola crisis, the lasting dysfunctioning of the health ministry, the prolonged absence of a health minister, and the failure to conduct a mid-term review of the NHDP. These uncertainties made partners aware of their interdependence and the need to work together to strengthen Togo’s health system. The decision-maker from the health ministry who was in charge of health planning also acknowledged the need for multisectoral collaboration. Her leadership led to the active mobilization of ministry stakeholders, as well as the local civil society, in a context that was nonetheless unconducive to its participation. WHO’s message on collaboration in Togo was especially convincing due to its symbolic capital [45] in the country. WHO played the role of boundary spanner, with the leadership of the WHO representative and Partnership’s team kick-starting collaboration between technical and financial partners.

Liberia is a country where we observed an unconducive environment. We noted a lack of interest in policy dialogue on aid coordination, despite the importance given to health sector coordination in policy documents and the gaps identified in the management of the Ebola crisis. Stakeholders were unaware of their interdependence, which can be explained by two factors. First, cooperation between the United States and Liberia is robust, unlike in the other countries of the study, and USAID plays a significant role in development aid. This situation, combined with the two countries’ shared history, gives the United States reliable symbolic power. As the Liberia government leading aid partner, the US and its agency are not much interested in participating in horizontal collaboration initiatives. Second, stakeholders were more concerned about the continuity of high-quality care and services following the Ebola crisis than aid coordination. Although the health sector was under significant internal pressure, including the need to improve coordination and the ministry’s objective to sign the Compact, external pressure was low, and concurrent short-term priorities were the primary concern. The policy dialogue supported by the Partnership was therefore marginal and perceived as imposed by WHO.

### The Partnership’s role in nurturing dialogue

Emerson et al. [16] recognize the importance of resources to build capacities for joint action in the framework of a collaborative governance device and to support collaboration dynamics. The Partnership provides financial and human resources to foster and support policy dialogue. The following demi-regularities shed light on the specific role of these resources once policy dialogue has been initiated.

#### Promoting principled engagement (demi-regularity 4)

*The Partnership promotes principled engagement among policy dialogue stakeholders (O) through facilitating knowledge generation and providing tailored technical expertise (I), which enable stakeholders to gain a shared understanding of issues and acknowledge the need for collective action (M), provided that they understand the policy dialogue process and see the added value of their contribution (C).*

We derive this demi-regularity from our observations in Togo, DRC, Niger, and Burkina Faso, where we were able to identify one or more of its components. In Niger, Togo, and Burkina Faso, knowledge generation to better understand the policy dialogue issues was an added value for most stakeholders. Such knowledge was generated in collaboration with other financial and technical partners or as part of the policy dialogue itself, such as in Togo and Niger. Combined with technical expertise, which was particularly present in Burkina Faso, this information led to the drafting of policy documents, which, in Niger and Togo, were viewed by participants as more realistic and less ambitious than previous ones, and which, in Burkina Faso, were considered to be of good quality and relevant. To sum up, accessing such knowledge generated mutual understanding (discovery). Also, the opportunity to share experiences and the need to reach consensus on what options they would propose and what choices and commitments they would make, led to a new way of producing policy documents (deliberation) based on negotiation and principled engagement.

Togo’s experience was the most successful in this regard: multisectoral policy dialogue enabled stakeholders to meet, share a certain amount of information and compare their experiences and perspectives on the issues identified during the 2012–2015 NHDP evaluation. Participating ministries and civil society also had the opportunity to appreciate their work’s repercussions within the health sector. Policy dialogue, to which stakeholders actively contributed by conducting the evaluation themselves, led to shared responsibility for future failures and mistakes. By participating in the process, they became aware of their contribution and responsibility for the failures encountered. Furthermore, since all actors were involved in a new and transparent evaluation process, the health ministry was obliged to assume its share of responsibility for its results. Joint evaluation, which involved representatives of other institutions and organizations, was thus seen as a way to monitor the health sector’s results and ensure their validity. This positive experience is the result of past policy dialogue initiatives supported by the Partnership since 2012, which paved the way for multistakeholder collaboration.

In the other countries, policy dialogue initiatives were not always well received by participants. For example, in DRC and Niger, some stakeholders felt their participation was instrumentalized or expressed their frustration. In DRC, one possible explanation for this reaction is that several policy dialogue initiatives on UHC took place simultaneously, and people participating in several of them may have felt confused. Another reason could have been the weak institutionalization of policy dialogue and insufficient leadership in the health ministry, which may have caused a lack of transparency in the policy dialogue process. In Niger, although policy dialogue on health planning seemed routine, the concertation spaces were dysfunctional, mostly because of high absenteeism among stakeholders. Besides, strategic and political decision-making processes did not seem well understood by stakeholders outside of the health ministry, which caused frustration. As the scientific literature corroborates, when policy dialogue is not institutionalized as a governance mechanism or guided by any leadership, participants may feel taken advantage of since they have a limited understanding of the process and are unaware of their contribution [15].

#### Motivation through collective leadership (demi-regularity 5)

*When health ministries are dynamic and engaged (C), the Partnership encourages stakeholders’ commitment to policy dialogue (O) by promoting collective leadership in key positions (M). Collective leadership increases participants’ involvement and motivation (O) owing to the symbolism associated with decision-makers’ hierarchical positions (M) and with reciprocity (M).*

This configuration originates from our observations in Togo, Niger, and Burkina Faso. In Togo, health planning policy dialogue benefitted from collective leadership. Collective leadership is a process in which several stakeholders play a separate but complementary role [57]. In Togo, such leadership arose from a fruitful collaboration between WHO, specifically the two Partnership full-time experts, and the health ministry, which saw value in the collaborative process and took the lead in policy dialogue. Collective leadership materialized in the strong engagement of several leading figures, including the health ministry’s decision-maker, WHO Representative, and the Partnership experts. Because of their hierarchical position in their organization, the health ministry decision-maker and WHO Representative had strong legitimacy. Their active, constructive, and continued involvement throughout the process made participants aware of the importance of policy dialogue. This involvement, combined with the Partnership experts’ continued support, motivated participants to engage in policy dialogue with determination, triggering both hierarchical and collaborative reciprocity [58, 59]. Hierarchical reciprocity was triggered for the health ministry decision-maker’s collaborators, while collaborative reciprocity was triggered for the WHO representative’s collaborators. Reciprocity explains why participants were actively involved and even surpassed themselves (for example, by working extra hours without pay, using their personal resources to conduct fieldwork). It prompted a positive feedback loop leading to strengthened collaboration between stakeholders. Niger and Burkina Faso are counterexamples since policy dialogue was less vigorous than in Togo. In Niger, health planning policy dialogue was embedded in an organizational routine that hampered momentum and change and involved few decision-makers. In Burkina Faso, policy dialogue on health financing was dynamic at the technical level but lacked high-level decision-makers, which delayed the process and lessened participation. As a result, WHO was more present to try and energize the process. WHO enduring presence explains why it seemed the main, if not sole, policy dialogue promoter to some stakeholders. In Burkina Faso, not only did the health ministry’s decision-makers lacked vigor, but WHO was also less involved than in Togo in policy dialogue at the strategic level, which could be owing to the fact there was no full-time Partnership expert to promote collaboration and policy dialogue within WHO.

### Policy dialogue dynamics

Despite the Partnership’s support, several challenges can reduce the Partnership and policy dialogue’s breadth. These challenges interfere with policy dialogue dynamics and affect policy dialogue’s institutionalization and sustainability. The following demi-regularities highlight the relation between these challenges and the development of policy dialogue as a collaborative governance mechanism over time.

#### Lack of ownership as a barrier to participation (demi-regularity 6)

*In the context of commodification of meeting opportunities (C), weak ownership of policy dialogue by health ministry decision-makers creates an adverse environment that discourages stakeholders (M) and reduces their participation (O), despite the Partnership’s support.*

Ensuring the continued participation of stakeholders has been the biggest challenge in policy dialogue. Participants were absent on a relatively regular basis in all the countries except for Togo, where stakeholders recognized collective leadership as the primary driver of their participation. In Niger, and to a lesser extent, in Burkina Faso, reduced participation was observed in the technical spheres of policy dialogue. Policy dialogue at a technical level usually involves experts from relevant ministries, national or international experts from development aid organizations, and sometimes civil society representatives. The irregular participation of ministerial collaborators in these technical spheres could be partially explained by the seminars phenomenon [38], which, related to the per diem culture and the habit of combining several representation mandates, have led to a commodification of meeting opportunities. The absence of ministerial collaborators prolonged the policy dialogue process, resulting in fewer external participants. Health ministries’ lack of ownership of policy dialogue could also explain the reduced participation of stakeholders. Several institutional shortcomings undermined policy dialogue at the strategic level. Policy dialogue at the strategic level usually brings together ministerial decision-makers, senior officials, international experts, representatives of international organizations, and sometimes civil society representatives. These deficiencies, such as irregular meetings, the lack of preparation for and follow-up of meetings, and the regular absence of key participants, reflected poor leadership and policy dialogue support, which in turn suggested a lack of interest and ownership. This situation occurred in Liberia, DRC, and Burkina Faso. In Burkina Faso and DRC, the discontinuity of mandates at key positions in the health ministries also impacted policy dialogue. These deficiencies prolonged and disrupted the flow of policy dialogue since they prevented decision-making. They created an adverse environment and contributed to institutional fatigue, a situation whereby stakeholders begin to lose sight of the purpose of their participation [60]. Such an environment led to a decrease in stakeholder participation. Furthermore, the health ministries’ weak ownership over policy dialogue prevented it from being institutionalized and implemented smoothly and transparently. This could reflect poorly on policy dialogue in the long term, particularly in contexts saturated with workshops, seminars, and other meetings. It also raises the question of whether policy dialogue is sustainable without external financing. The examples of Togo, where the health ministry’s decision-maker took charge of policy dialogue, and Burkina Faso, where the arrival of a more engaged decision-maker made it possible to reinitiate policy dialogue on health financing at the strategic level, demonstrated that ministerial leadership was needed for ownership to arise.

#### The institutionalization of multisectoral collaboration (demi-regularity 7)

*In a context of collective leadership (C), full-time international experts (I) promote ownership over policy dialogue processes (O) by responding to the needs of their ministerial counterparts and by helping them to establish and monitor policy dialogue (M), which contributes to the institutionalization of multisectoral collaboration (O).*

We derive this semi-regularity, which sheds light on technical assistance’s long-term role, from the six cases where we were able to identify one or more of its components. The presence of a short-term expert and knowledge generation may strengthen capacities for joint action in policy dialogue. However, this only concerns the technical aspects of health financing (for example, in Burkina Faso and DRC) and health planning (in Niger and Togo). When it comes to the institutionalization of multisectoral collaborative governance mechanisms, health ministry decision-makers must take ownership of such mechanisms at the strategic level. Weak ownership of policy dialogue, as observed in Burkina Faso, DRC, Niger, and Liberia, is known to cause technical assistance programs to fail [56]. The Partnership’s different modes (i.e., a light mode involving a part-time international expert and full mode involving a full-time international expert), can partially explain this challenge. The role of international experts is to promote multisectoral collaboration in health ministries, with the risk that the latter may view policy dialogue as merely another version of the seminars phenomenon. Their role is also to help ministries gain the expertise to establish and provide impetus to policy dialogue. In Burkina Faso and Niger, the unavailability of a full-time international expert, turnover of decision-makers, and limited interest in policy dialogue's topic may account for the decision-makers' weak ownership. In Cabo Verde, WHO and the health ministry used external consultants in regional health planning and had no direct support role. As a result, ownership of the process by ministerial actors was weak. Of all the countries that benefitted from a full-time expert, Togo is the only country where the health ministry's decision-maker recognized the added value of policy dialogue and took ownership of the process. We also noticed that, in Togo, the Partnership’s experts and the decision-maker trusted one another. Their relationship had grown over time owing to both WHO office's geographical proximity and the full availability of the policy dialogue experts. Three mechanisms lead to collective action: reciprocity, reliability, and trust (Ostrom [61], cited in Thomson and Perry [59]). These mechanisms manifested in the partnership between WHO and Togo's health ministry. In DRC and Liberia, there was no ownership over policy dialogue by health ministries, despite the presence of full-time experts. Our research points to two main reasons: the limited interest in policy dialogue in both countries, especially at the strategic level, and the lack of synergy in WHO country offices in promoting multisectoral collaboration and assuming their role as boundary spanners. Collective leadership never emerged, and the message on multisectoral collaboration was much less clear than in Togo.

#### The challenge of building confidence in collective action (demi-regularity 8)

*In contexts where the health ministry demonstrates weak leadership (C), policy dialogue is unlikely to foster collaboration of stakeholders for the implementation of collective decisions (O) since policy dialogue participants lack confidence in their capacity for joint action and the ministry’s abilities to take its stewardship role (M).*

This demi-regularity originates from our observations in Togo, Liberia, and DRC, where, throughout the study, informants addressed the challenge of implementing collective decisions and proposals for action set out in the documents drafted during policy dialogue. Policy dialogue systematically leads to policy documents regardless of stakeholder collaboration, as observed in all the countries. However, drafting these documents is just the beginning of collaborative action, as participants confirmed. The goal is to implement the policy documents’ recommendations or guidelines and to coordinate the stakeholders. As stated in the intervention theory, ownership of policy documents, which originates from the collaborative process of policy dialogue, should encourage participants to move to implementation [20]. In our study, however, informants were pessimistic or had an attitude of resignation when talking about actors’ collaborative capacities in the health sector. They lacked confidence in institutional partners’ abilities, including governments and health ministries, to carry out their role as a guide or steward and assure the next steps of policy dialogue. In Liberia and Togo, informants pinpointed the challenge of comprehending the policy documents drafting process as a step towards public action. Such documents were instead seen as the policy dialogue's end goals, allowing stakeholders – including the ministerial collaborators responsible for producing such documents – to "tick a box". The health sector's weak governance was a clear topic of discussion. Informants noticed that health sector stakeholders usually failed to meet commitments. It was especially true in Togo, where the 2012–2015 NHDP evaluation results led to disappointment among stakeholders. The NHDP had created high expectations since all stakeholders in the sector had made commitments, following the Compact’s signature, which was an outcome of policy dialogue. Informants also called into question the openness to change among development aid actors and institutional stakeholders. Path dependency can explain such a sentiment: in French-speaking countries in Western Africa, stakeholders seem to have found their place in the national arena, especially since "daily governance" tends to prevent collective action [35]. As a result, even though policy dialogue in specific contexts motivate participants and promotes principled engagement, which is a driver of continued participation, the lack of confidence in joint action could make policy dialogue opportunities seem less attractive over time [62], especially if the challenges of implementation deceive expectations.

## Discussion and conclusion

In this section, we will first highlight the challenges of evaluating initiatives aimed at strengthening governance capacity of national institutions, such as the Partnership, and suggest ways forward for researchers. We will then make recommendations for development aid actors interested in policy dialogue as a collaborative health governance mechanism.

### Understanding collaborative governance to highlight the contribution of governance capacity building initiatives

**Fig. 4:**
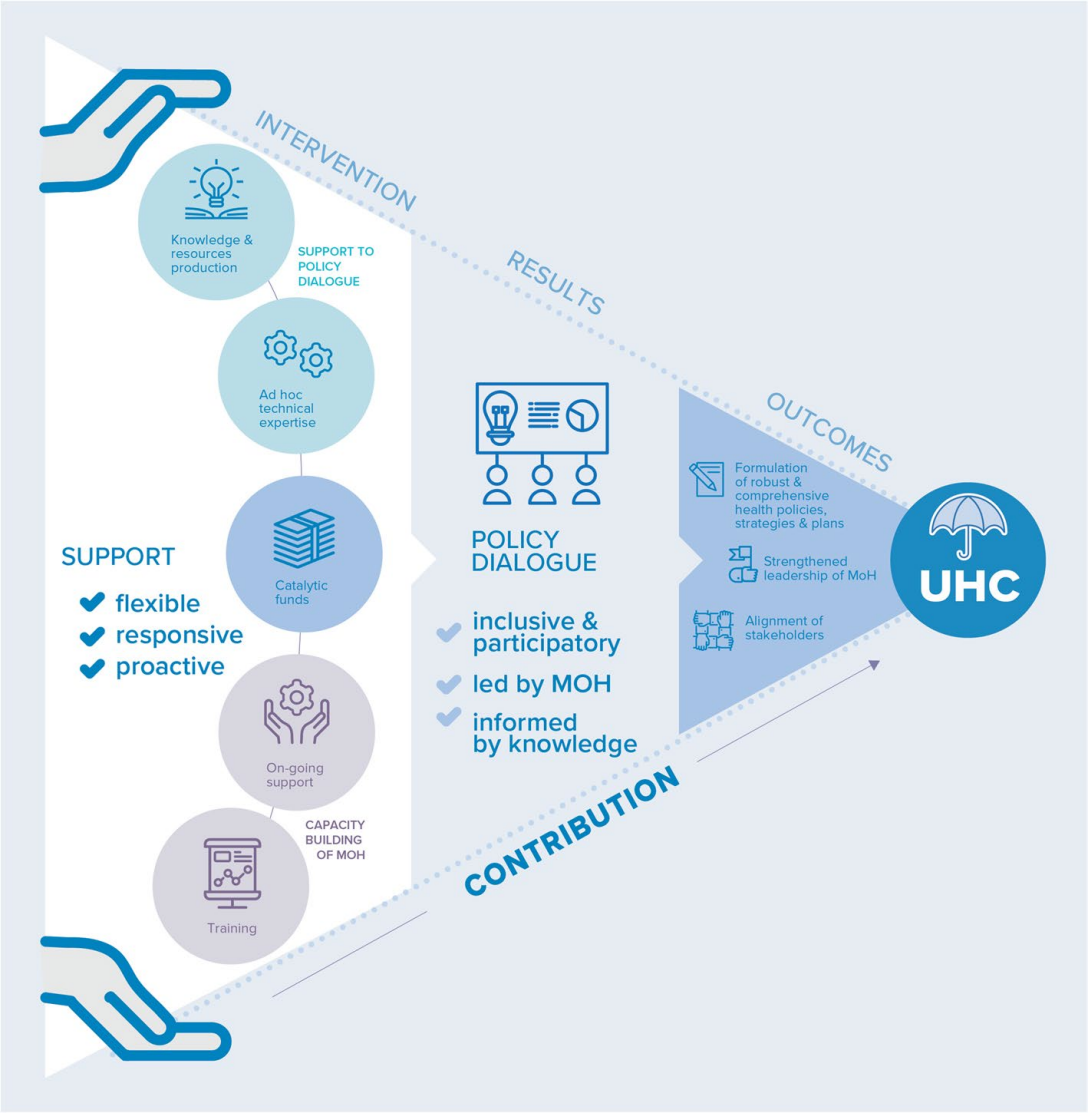
The Partnership theory

In the international development arena, evaluating programs that aim to strengthen national institutions’ capacities, such as the Partnership, involve two significant challenges. First, organizations that receive external funding, such as WHO, are accountable and have to demonstrate concrete results. This challenge especially applies to the health sector, where evaluation efforts are usually focused on measuring the direct outcomes of interventions, most of which target diseases, and where priority is placed on high-impact interventions. Standard research designs lack, however, validity when looking at capacity building initiatives, especially when such initiatives target governance. The complexity and the high number of interactions between such programs and contextual features, the multitude of actors involved and reasoning behind their actions, and the long duration of governance processes, are among the many reasons why it is problematic to identify a direct and strong association between the program and its expected outcomes. It thus becomes essential that stakeholders tasked with monitoring and achieving those outcomes appropriately define outcomes, in a specific context. This requirement leads to the second challenge: identifying and agreeing on an accountability ceiling [63]. From this threshold, an organization may refuse to take sole responsibility in achieving long-term and multifactorial outcomes. In our research, the intervention theories of the Partnership have made it possible to detach from UHC, which is a long-term and societal outcome, and identify WHO’s accountability ceiling. This exercise enabled us to focus on the Partnership's actions to initiate and structure policy dialogue as a collaborative governance mechanism (Fig. 4). Understanding collaborative governance became the main object of our study in order to expose the processes and contributions of the Partnership.

Based on our experience, we recommend that researchers consider two points when assessing the outcomes of programs supporting capacity building for collaborative governance. First, researchers should investigate sustainability, defined as the routinization of stable organizational procedures and behaviors [64]. Sustainability implies a balance between the governance mechanism and the governance culture [64]. Evaluations should thus focus on whether programs helped foster a culture of collaborative governance by building the necessary capacities for joint action. Researchers should also evaluate the content of policy documents, which are concrete and observable deliverables, and the proposed implementation mechanisms for follow-up and accountability. Second, research looking into systemic changes or investigating whether public actions, such as measures towards UHC in our case, were implemented and produced outcomes requires long-term investigations. This time is needed for collaborative governance mechanisms to take effect and for the culture of collaboration to percolate through other branches of public administration and stakeholders, to produce what Emerson refers to as the sustaining capacity for joint action [16]. Assessing joint action and its outcomes involves, among other things, considering transitory outcomes and their sequence that mark the progression of the action up to its outcomes [65].

### Supporting collaborative health governance in LMIC

Collaborative governance faces great challenges in LMIC. Political instability, limited resources, and hierarchal systems dominated by clientelism [19, 35] complicate collaboration dynamics and efforts toward a shared vision of accountability [19]. Our realist evaluation of the Partnership in six countries shows mixed results regarding collaborative governance and points to similar challenges that commonly undermine technical assistance programs. In contexts of strong international mobilization for UHC, WHO’s actions benefitted from synergies between the Partnership and other international stakeholders’ resources. This contributed to the mobilization of national actors to draft key policy documents for UHC, provided that such documents were a national priority and were supported by health ministries with strong political will. At the same time, keeping stakeholders involved in policy dialogue was a challenge, mainly when institutional arrangements were inadequate, and there were poor leadership and confidence in the collective capacity to move to action. In countries where policy dialogue was passive, there was a lack of collective leadership and shared responsibility for policy dialogue. The process was left into the health ministry's hands, whereas it was the most in need of support. In this context, despite the support of WHO and other partners, discussions and deliberations remained at the technical level, and strategic decisions were delayed. Ultimately, the challenge of taking action and lack of confidence among stakeholders in the policy dialogue’s impacts call into question the sustainability of these mechanisms. From these findings and challenges, we suggest four avenues for action.

First, WHO, or any organization aiming at fostering policy dialogue through capacity building in LMIC, need to adapt to the environments in which they operate. For initiating policy dialogue, experts and technical assistants must answer stakeholders’ interests or needs for support. Although necessary, alignment between support and needs alone is not enough. A combination of other drivers is needed. WHO has to provide the leadership towards multisectoral collaboration and the inclusion of civil society. It must also help stakeholders to become aware of their interdependence in confronting health sector challenges and uncertainties. WHO should also carefully analyze internal and external pressures and tailor its support and advocacy.

Second, to enhance stakeholders’ principled engagement and support capacities for joint action through policy dialogue, WHO must strengthen three resources that have demonstrated their added value in the Partnership: 1) policy dialogue international experts who support health ministries and promote inclusivity and multisectoral collaboration; 2) financial support for organizing meetings that support exchanges between stakeholders and the joint drafting of policy documents; and 3) funding for activities that generate knowledge, nurture exchange, enhance stakeholders’ competencies and create mutual understanding. In addition, in order to rally ministerial stakeholders, WHO must consider the per diem culture and assess the interest surrounding both the policy dialogue's topic and terms and conditions (inclusive and multisectoral). It must also consider power dynamics, when involving other development aid actors, and should ensure organizations that have significant economic and social capital are keen in collaborating. WHO in countries must also take better advantage of the international context, which is favorable to both UHC and health systems strengthening, to vigorously promote multisectoral collaboration, especially as national regulations allow for multisectoral collaboration.

Third, to support international experts’ efforts, WHO must ensure that they have direct relationships with dynamic decision-makers in health ministries, not only with technical staff. International experts should be facilitators: they should promote collective leadership and support proactive decision-makers. They should aim to institutionalize policy dialogue, ensuring that it is transparent and clear to participants to avoid disappointment and misunderstanding, and turn the policy dialogue into a positive experience.

Fourth, to strengthen the institutionalization of policy dialogue as a governance mechanism, WHO should ensure that health ministry decision-makers take ownership of policy dialogue, understand its added value, and approve of this form of collaboration, while respecting institutional arrangements. In this regard, the support provided by full-time international experts is essential in promoting a cross-cutting approach to policy dialogue, ensuring close follow-up, seizing opportunities to reinvigorate the process, and building trusting relationships with health ministries. In order to mobilize development aid actors and galvanize health ministries, WHO should increase synergies in promoting multisectoral collaboration for UHC in countries at the technical, strategic, and policy levels. There is also a need to build confidence in capacities for joint action, which seems to have eroded in a context of competition over resources and slow progress. When policy dialogue increases motivation, WHO, health ministries, and development aid partners should strive to maintain this motivation and ensure that recommendations are implemented.

Given the challenges in strengthening collaborative governance at the central level, now could be a good time to create synergies with local governance and citizens' initiatives. According to Olivier de Sardan [35], well-performing areas ("îlots de fonctionnalité") at the lower levels of States should be strengthened by applying real standards of practice and professional cultures, rather than imported bureaucratic rules. This would help to establish a culture of collaborative governance. Improving these processes certainly requires leaders and international experts who uphold and defend these values; however, these defenders or champions, who are “notable exceptions” [35], can only prove their capabilities in a supportive systemic context. In that respect, Greer et al. refer to the need to adopt a structural vision of governance, since “[i]t is too simple to wait for a great leader, and leaders can often disappoint” [57]. More concretely, it is essential to create conditions for a culture of collaboration to emerge and maintain in order to strengthen health systems governance and achieve UHC. These conditions include building collaborative governance at the many levels of public administration, ensuring that processes are transparent and understandable in order to promote stakeholders’ involvement, and engaging in good faith by supporting collaboration not only as a tool but also as the cornerstone of effective and sustainable health systems enabling UHC.

Discourse on collaboration continues to be stronger at the international level than on the ground; more time is needed for it to gain momentum in countries and for the message to percolate among development aid actors. Therefore, the Partnership’s added value primarily depends on context and forms part of a collaborative approach that requires all stakeholders’ goodwill and, as a minimum, a supportive environment. It is the interaction between the Partnership, particularly the quality of its content and the form of its technical assistance, and favorable contextual elements that will make it possible to achieve a certain number of positive outcomes.

## Data Availability

The datasets generated and analysed during the current study are not publicly available due to limitations of ethical approval involving the participants’ data and anonymity. Case studies may be available from the corresponding author with the agreement of WHO.

## Abbreviations

CIMO: Context-intervention-mechanism-outcome
DRC: Democratic Republic of the Congo
LMIC: Low- and middle-income countries
NHDP: National health development plan
UHC: Universal Health Coverage
USAID: United States Agency for International Development
WHO: World Health Organization
